# Circulating Permeability Factors in Focal Segmental Glomerulosclerosis: *In* *Vitro* Detection

**DOI:** 10.1016/j.ekir.2022.09.014

**Published:** 2022-09-20

**Authors:** Susan T. Veissi, Bart Smeets, Joanna A.E. van Wijk, René Classens, Thea J.A. M. van der Velden, Annelies Jeronimus-Klaasen, Floor Veltkamp, E.M. Mak – Nienhuis, William Morello, Giovanni Montini, Antonia H.M. Bouts, Lambertus P.W. J. van den Heuvel, Michiel F. Schreuder

**Affiliations:** 1 Department of Pediatric Nephrology, Amalia Children’s Hospital, Radboud University Medical Center, Radboud Institute for Molecular Life Sciences, Nijmegen, the Netherlands; 2 Department of Pathology, Radboud university Medical Center, Radboud Institute for Molecular Life Sciences, Nijmegen, the Netherlands; 3 Department of Paediatric Nephrology, Emma Children’s Hospital, Amsterdam University Medical Center, Amsterdam, the Netherlands; 4 Department of Pediatric Nephrology, Dialysis and Transplant Unit, Foundation IRCCS Cà Granda, IRCCS Ospedale Maggiore Policlinico, Milan, Italy; 5 Department of Laboratory Medicine, Radboud University Medical Center, Radboud Institute for Molecular Life Sciences, Nijmegen, the Netherlands; 6 Department of Development and Regeneration, University Hospital Leuven, Leuven, Belgium

**Keywords:** actin cytoskeleton damage, circulating permeability factor, focal segmental glomerulosclerosis, nephrotic syndrome, podocytes, reactive oxygen species

## Abstract

**Introduction:**

The recurrence of proteinuria after kidney transplantation in patients with focal segmental glomerulosclerosis (FSGS) is considered proof of the presence of circulating permeability factors (CPFs). The aim of this study is to demonstrate the presence of plasma CPFs using series of *in vitro* assays.

**Methods:**

Podocytes and endothelial cells (glomerular microvascular endothelial cells [GMVECs]) were incubated with plasma from FSGS patients with presumed CPFs in relapse and remission and from steroid-resistant nephrotic syndrome (SRNS), steroid-sensitive nephrotic syndrome (SSNS), membranous nephropathy (MN), and healthy controls (hCtrls). Cell viability, podocyte actin cytoskeleton architecture, and reactive oxygen species (ROS) formation with or without ROS scavenger were investigated by Cell Counting Kit-8 assay, immunofluorescence staining, and CM-H2DCFDA probing, respectively.

**Results:**

Presumed CPF-containing plasma causes a series of events in podocytes but not in GMVECs. These events include actin cytoskeleton rearrangement and excessive formation of ROS, which results in podocyte loss. These effects were solely observed in response to CPF plasma collected during relapse, but not in response to plasma of hCtrls, or patients with SRNS, SSNS, and MN. The copresence of dimethylthiourea, a scavenger of ROS, abolished the aforementioned effects of CPF plasma.

**Conclusion:**

We provide a panel of *in vitro* bioassays to measure podocyte injury and predict the presence of CPFs in plasma of patients with nephrotic syndrome (NS), providing a new framework for monitoring CPF activity that may contribute to future NS diagnostics or used for disease monitoring purposes. Moreover, our findings suggest that the inhibition of ROS formation or facilitating rapid ROS scavenging may exert beneficial effects in patients with CPFs.

## Graphical abstract

**Figure:**
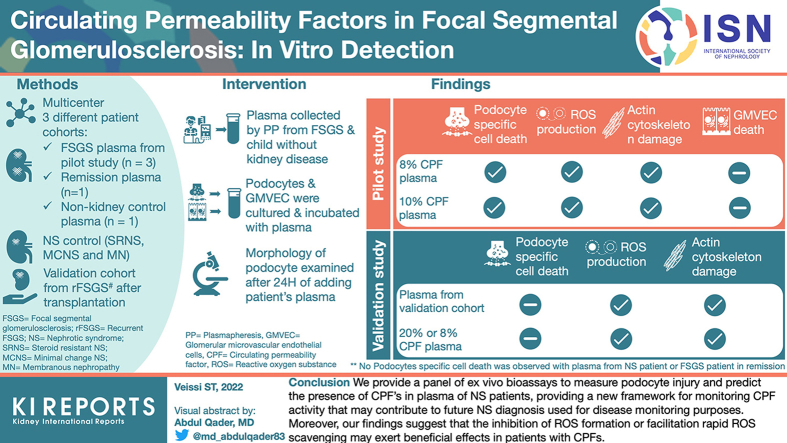

NS is diagnosed in the presence of the triad of proteinuria, hypoalbuminemia, and edema. Most children presenting with NS will have minimal change NS (MCNS), and show a steroid-sensitive course of the disease. Nevertheless, some NS patients will be steroid resistant (SRNS), and a kidney biopsy may show either MCNS or FSGS, which are considered to present 2 ends of a spectrum.^1^

FSGS is a histopathologic pattern of injury and may be caused by several factors, including plasma-derived CPFs. These factors are thought to cause podocyte injury, the hallmark of FSGS, which leads to the loss of podocytes and consequently permeabilization of the glomerular filtration barrier.^2^^,^^3^ Evidence for CPFs in FSGS includes remission after plasmapheresis (PP) or immunoadsorption treatment, the development of proteinuria after plasma donation, the fast recurrence of FSGS after kidney transplantation, and functional recovery of a kidney allograft after explantation from a FSGS patient into a non-FSGS patient.^4^, ^5^, ^6^, ^7^ This has fueled research interest in finding the CPF that causes FSGS because this may directly impact the treatment of FSGS patients.^8^, ^9^, ^10^ Unfortunately, this search has not yet led to the unambiguous designation of a candidate molecule. Instead, a wide variety of candidate molecules (i.e*.* soluble urokinase plasminogen activator receptor, cardiotrophin-like cytokine factor-1, and hemopexin) have been described.^1^^,^^11^ This may suggest CPFs are most likely various different etiopathogenic agents. Therefore, focus of recent research has shifted toward the development of noninvasive prognostic bioassays to identify patients with plasma-derived CPF regardless of the identity of a specific CPF.

It is highly important to distinguish between NS patients with CPFs and other forms of NS because patients with presumed CPFs have shown to have a high likelihood of NS recurrence after kidney transplantation, but also to respond to extracorporeal therapies such as plasma exchange therapy, immunoadsorption, and low-density lipoprotein apheresis. Therefore, using podocyte injury as an experimental readout, we aim to explore novel *in vitro* bioassays that can predict the presence of CPFs, irrespective of its nature, in the plasma of patients with suspected CPFs.

## Methods

### Patient Material

This study was conducted in accordance with the recommendations of the appropriate version of the Helsinki Declaration. Informed consent from all subjects was obtained. Three different patient cohorts, namely the pilot study cohort, the NS control cohort, and the validation cohort were used for this study (See Table 1 for relevant clinical characteristics). The patient samples with presumed CPF used for this study were defined as a patient with native kidneys with SRNS that showed ongoing NS with kidney function deterioration despite treatment with steroids, cyclosporine, and rituximab, who were treated with PP that resulted in massive improvement of proteinuria and kidney function. This supports the presence of a plasma-derived CPF.

**Table 1:** Patient characteristics. Clinical characteristics of the patients included in this study

Sample	Gender (M/F)	Age	eGFR (ml/min/1.73 m^2^)	P/C ratio (g/10 mmol)	Albumin (g/l)	Edema	PP (1/2)	Transplant	Mutation	Diagnosis (Biopsy)
The pilot cohort										
Ctrl.	F	11	185	0.20	35	None	1	No	NA	NMO (No)
Act. 1	F	7	77	19	20	Some	1	Yes	None	FSGS,active (FSGS)
Act. 2	M	15	34	8.83	23	Some	1	No	None	FSGS, active (FSGS)
Rem.	M	16	73	0.61	28	None	20	No	None	FSGS, remission
The NS control cohort										
SRNS 1.	M	2	99	23.13	18	Yes	0	No	None	SRNS first presentation (MCNS)
SRNS 2.	F	4	138	5.61	31	None	0	No	None	SRNS first presentation (FSGS)
MCNS Act 1.	M	14	90	8.32	10	Yes	0	No	None	MCNS first presentation (No)
MCNS Act 2.	M	3	-	(559 mg/day)	21	Yes	0	No	None	MCNS first presentation (No)
MCNS Act 3.	M	3	105	27.82	12	Yes	0	No	None	MCNS first presentation (No)
MCNS Rem 1.	M	14	114	0.08	38	None	0	No	None	MCNS remission (No)
MCNS Rem 2.	M	3	209	0.95	45	None	0	No	None	MCNS remission (No)
MCNS Rem 3.	M	3	120	0.62	41	None	0	No	None	MCNS remission (No)
MN 1.	F	50	42.9	0.49	32	Some	0	No	None	MN active (NA)
MN 2.	M	37	117.8	0.36	25	Some	0	No	None	MN active (NA)
The validation cohort										
Act 3.	M	4	18	15	19	Severe	1	No	None	FSGS active (FSGS)
MI-1	F	17	37	12.5	3.1	None	2	Yes	None	rFSGS (NA)
MI-2	F	21	67	(3780 mg/day)	4.0	None	2	Yes	None	rFSGS (NA)
MI-3	F	11	110	12	3.3	None	2	Yes (2x)	None	rFSGS (NA)

Act. 1/2/3, active 1/2/3; Ctrl, control; eGFR, estimated glomerular filtration rate; F, female; FSGS, focal segmental glomerulosclerosis; M, male; MI-1/2/3, rFSGS samples obtained from Milan Italy; MCNS, minimal change nephrotic syndrome; MN, membranous nephropathy; NA; not available; NMO, neuromyelitis optica; P/C ratio, urinary protein-creatinine ratio; PP, plasmapheresis; PP1, plasmapheresis in native kidney; PP2, plasmapheresis after kidney transplantation due to relapse; Rem, remission; rFSGS, recurrence focal segmental glomerulosclerosis; SRNS, steroid-resistant nephrotic syndrome.

PP was initiated based on the decision from the treating physician and with informed consent from the patient (and according to age, from his or her parents) using the Prismaflex system (Baxter International) with a TPE1000 or a TPE2000 filter, based on the size of the patient. From the first PP session, the first 500 ml was collected, aliquoted and stored as “active” FSGS plasma. When patients were responsive to the PP treatment, the first 500 ml of the final PP session was also stored and used as “remission” FSGS plasma. Aliquoted samples used in experiments did not exceed 5 freeze-thaw cycles.

Three patients with SRNS admitted to the Radboud University Medical Center that were included in this study underwent a kidney biopsy. Electron microscopy analyses were performed using routine protocols in our diagnostic facility.

PP from a child without kidney disease was collected according to the PP protocol above and used as a nonkidney control in our study. Serum and lithium heparin plasma samples from patients with SRNS, MCNS, and MN were obtained and used as NS control samples in our experiments. Furthermore, a healthy control pool plasma was obtained from 8 healthy adult volunteers. Exclusion criteria for this control group included fever, bacterial or viral infection in the past 2 weeks, chronic illness, inborn or acquired immune disorders, and the use of immunosuppressive drugs.

To validate our assays, PP samples from 1 active patient with presumed CPF and 3 recurrent FSGS (rFSGS) patients were included. The rFSGS PP samples were obtained from patients with relapses after transplantation.

### Cell Culture

Conditionally immortalized human podocytes (AB 8/13) were kindly provided by Professor Saleem (University of Bristol, UK).^12^ Podocytes were grown under permissive condition at 33 °C with 5% carbon dioxide in complete medium (CM) consisting of Roswell Park Memorial Institute -1640 medium (Sigma), supplemented with 10% fetal bovine serum (FBS; Gibco), 0.01% insulin-transferrin-sodium selenite (ITS; Sigma), and 1% penicillin-streptomycin (Gibco). Podocytes were differentiated at 37 °C with 5% carbon dioxide during 7 to 10 days in CM. In selected experiments, primary human GMVECs were used as well. GMVECs were obtained and cultured as described previously.^13^ Differentiated podocytes and GMVECs were incubated with varying concentrations of patient plasma. Patient plasma was in all cases supplemented with FBS to retrieve 10% plasma in all experiments.

### Cell Viability Assay

Confluent monolayer of podocytes or GMVECs (10,000 cells/well) were seeded on clear 96-well tissue culture plate (Catalog #3596 Corning). Cells were exposed to different concentrations of patient plasma for 24 hours. Cell viability was assessed by conventional light microscopy and with Cell Counting Kit-8 (CCK-8; Sigma) according to the manufacturer’s instructions. In brief, 10 μl of the Cell Counting Kit-8 solution was added to each culture condition and incubated for 4 hours at 37 °C. Absorbance was measured at 450 nm with the Victor 3 V Multilabel Plate reader (PerkinElmer).

### Immunofluorescence Imaging

Confluent monolayers of differentiated podocytes (10,000 cells/well) were seeded on black 96-well tissue culture plate (Greiner Bio-One). After exposure to patient plasma for 6 hours, cells were fixed with 4% paraformaldehyde (Sigma) for 15 minutes at room temperature and subsequently washed with Hank’s balanced salt solution (HBSS; Gibco). Cells were incubated in a blocking solution (1ml FBS + 1.0 g bovine serum albumin + 50 μl Tween20 in 50 ml 1× Hank’s balanced salt solution) for 15 minutes at room temperature. Alexa Fluor 488 Phalloidin (1:40 in phosphate buffered saline containing 0.2% Triton X-100; Catalog #A12379 Invitrogen) was used. DNA was stained with 4′,6-diamidino-2-phenylindole (1:500) for 5 minutes at room temperature. Cells were viewed with a Zeiss LSM880 laser scanning microscopy (Zeiss) using standard filter sets. Images were processed with Adobe Photoshop and signal intensities were quantified with histogram plots.

### ROS Measurement

The production of intracellular ROS was measured using the fluorescence probe reagent CM-H2DCFDA (Invitrogen). In brief, confluent monolayers of differentiated podocytes or GMVECs (10,000 cells/well) were seeded on black 96-well tissue culture plate (Greiner Bio-One). Cells were starved overnight preceding experiments with patient plasma at concentrations of 8% or 20%. Before exposure to patient plasma, cells were loaded for 30 minutes with 10 μM CM-H2DCFDA dissolved in Hank’s balanced salt solution-10 mM HEPES. ROS formation was measured by fluorometry (excitation/emission at 485/535 nm) with the Victor 3 V Multilabel Plate reader (PerkinElmer). In selected experiments, cells were pretreated for 1 hour with the ROS scavenger dimethylthiourea (DMTU; 10 mM, Sigma). Positive control conditions with 50 μM hydrogen peroxide were performed in all experiments.

### Statistical Analysis

Data are reported as the means ± standard error of the mean of at least 3 experiments. Significance was determined by *t*-test or 1-way analysis of variance followed by Bonferroni correction. *P* values less than 0.05 were considered statistically significant. All graphs were made using GraphPad Prism software version 5 for Windows (GraphPad Software, San Diego, CA, USA).

## Results

### Patients

**Figure 1:**
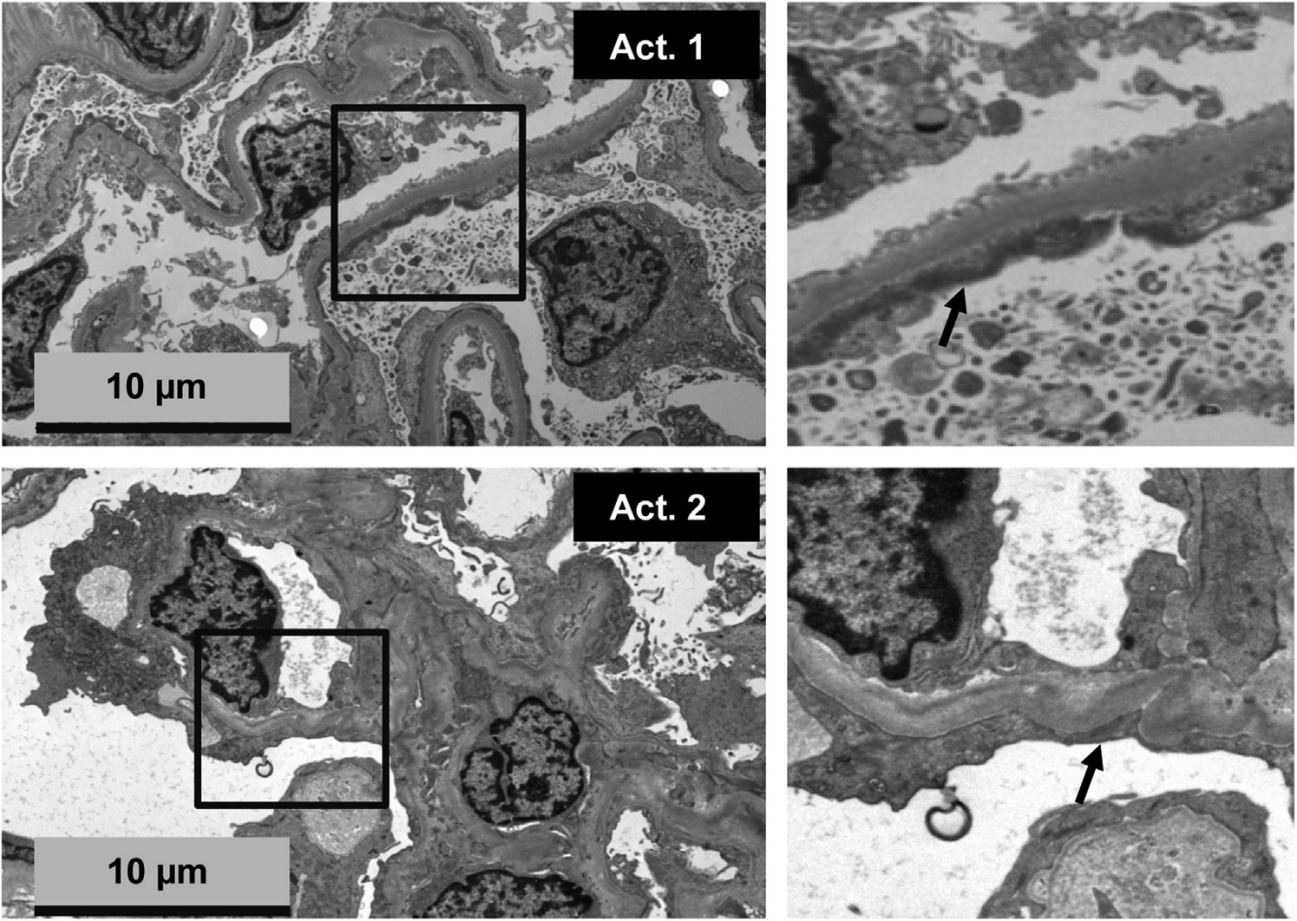
Kidney biopsy slices from FSGS patients. Representative electron microscopy images of kidney biopsies from 2 FSGS patients with active disease (Act. 1, Act. 2) showing hallmark feature of FSGS (i.e., podocyte foot effacement, arrows). Scale bars: 10 μm. FSGS; focal segmental glomerulosclerosis.

Four samples from 3 patients referred to the Radboud University Medical Center (Radboudumc) were included into the pilot study (Table 1). Two patients were diagnosed with active FSGS with presumed CPFs (active [Act.]1 and Act. 2), and from the second patient (Act. 2), a remission sample was available as well. Subsequently, 1 nonkidney control patient diagnosed with neuromyelitis optica having autoantibodies directed against myelin oligodendrocyte glycoprotein was included, because this patient was treated with the same PP protocol as the FSGS patients mentioned above. Kidney function, proteinuria (protein-creatinine ratio), serum albumin, and the presence of edema was documented. As depicted in Figure 1, hallmark features of FSGS such as sclerosis and podocyte foot effacement were seen in the kidney biopsies of the patients with FSGS (Act. 1 and Act. 2). The NS control group samples were obtained from 2 patients with SRNS at first presentation, 3 patients with MCNS at first presentation and their paired remission sample, and 2 patients with MN. All of the first presentation and active state samples were samples from patients with heavy proteinuria and hypoalbuminaemia. Remission samples were defined by absence of proteinuria for 3 days within 4 weeks of steroid treatment (Table 1). One patient with SRNS showed MCNS on kidney biopsy, whereas the second patient was diagnosed with FSGS on biopsy. Both showed remission of their NS with additional immunosuppressive therapy. Finally, the validation patient cohort, which consisted of a boy with active FSGS (Act. 3; admitted to the Radboudumc), who later showed a relapse after kidney transplantation, and 3 patients with rFSGS (Milan Italy-1, Milan Italy-2, Milan Italy-3) between 11 and 21 years of age.

### Active FSGS Patient Plasma Causes Podocyte-specific Cell Death

**Figure 2:**
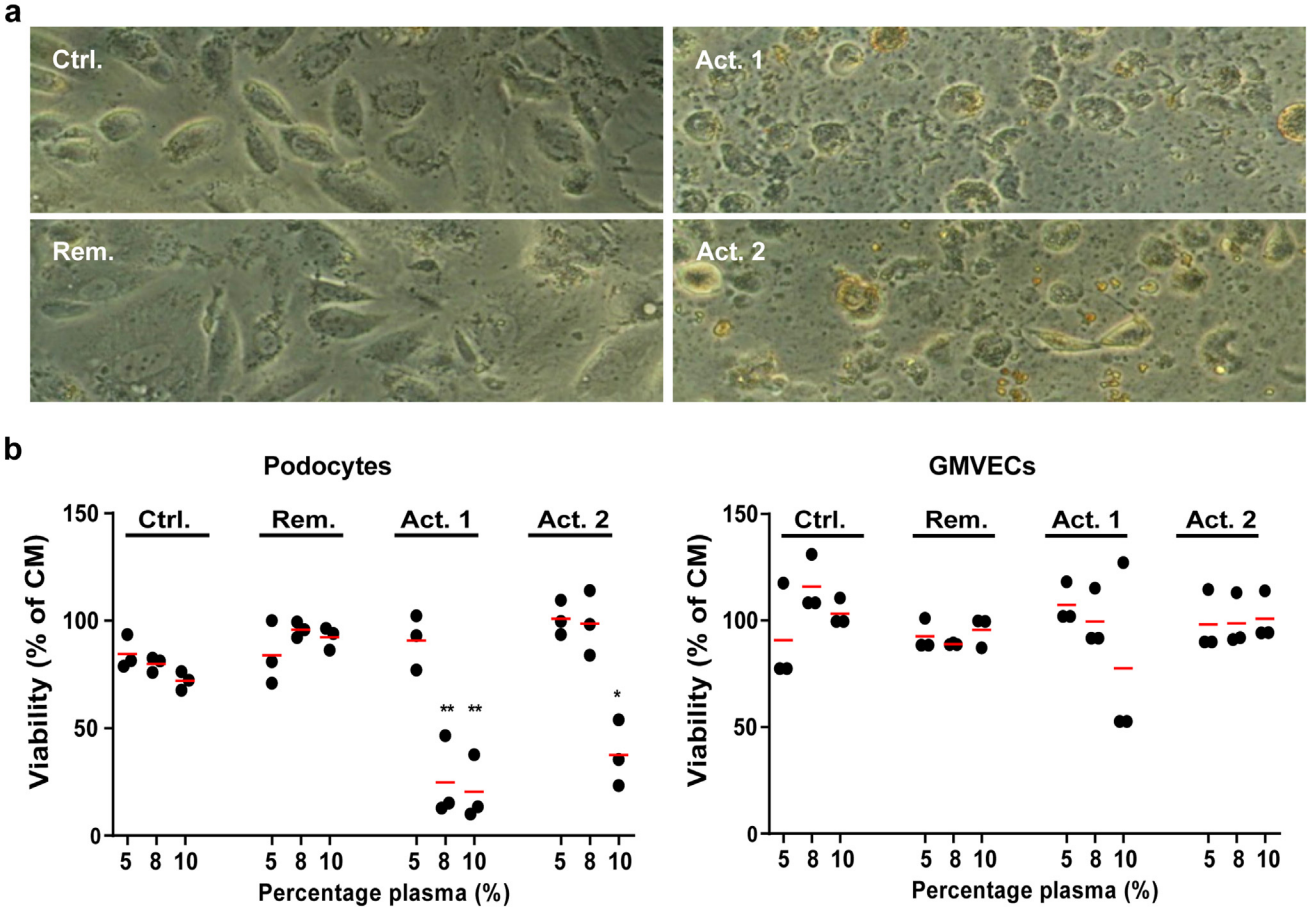
Active FSGS patient plasma causes podocyte-specific cell death. (a) Conventional light microscopy analyses reveals morphological alterations (detaching cells, membrane blebbing, and debris) suggestive of cell death after 24 hours exposure to 10% active FSGS patient plasma (Act. 1, Act. 2). In response to 10 % plasma from the control patient (Ctrl) or the FSGS patient in remission (Rem), these effects were not seen. (b) CCK-8 assay demonstrating that podocyte death in response to active FSGS patient plasma is concentration-dependent (left figure; AB 8/13 passage 37/38/39 [N = 3]). In contrast to podocytes, cell death is not detected in primary human glomerular microvascular endothelial cells (GMVECs passage 5/6/7) after exposure to patient plasma (right figure, N = 3). ∗*P* < 0.05, ∗∗*P* < 0.01 compared to corresponding plasma concentration of the podocyte complete medium. CCK-8, Cell Counting Kit-8; FSGS, focal segmental glomerulosclerosis.

We first investigated the morphology of podocytes using conventional light microscopy after 24 hours of exposure to 10% patient plasma. We observed drastic changes in podocyte cell morphology when plasma of active FSGS patients with CPF was present (Figure 2a). These morphological changes were not observed in the presence of plasma derived from the control patient or the FSGS patient that was in remission. Morphological changes consisted of the folllowing: (i) rounding of podocytes, (ii) membrane blebbing, and (iii) full cell detachment, all highly suggestive of (apoptotic) cell death. To confirm that the observed morphological changes were the result of cell death, we next performed a Cell Counting Kit-8 assay. This indeed showed podocyte cell death in response to CPF-containing plasma (Act. 1 and Act. 2) after 24 hours at a concentration of 8% or 10% (Figure 2b, left panel). Cell death in response to CPF-containing plasma from patients with active FSGS was concentration-dependent, because lowering the concentration of plasma to 5% prevented podocyte death. Interestingly, similar concentrations of plasma in experiments with primary GMVECs did not cause significant cell death, indicating that CPF-containing plasma of patients with active FSGS is specifically damaging to podocytes (Figure 2b, right panel).

### FSGS Patient Plasma With Presumed CPF Damages the Podocyte’s Actin Cytoskeleton

**Figure 3:**
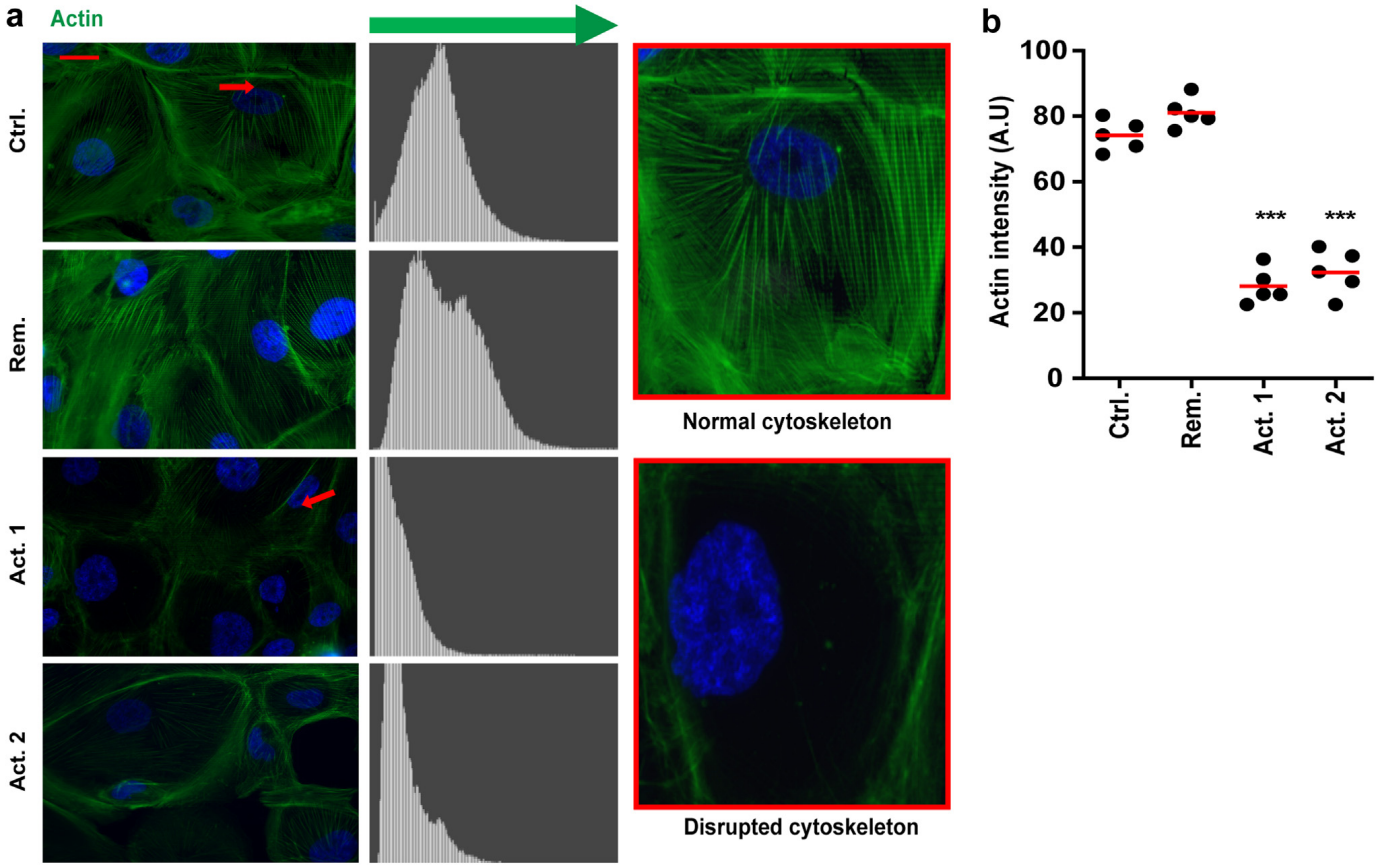
Active FSGS patient plasma containing CPF disrupts the podocyte’s actin cytoskeleton. (a) Representative immunofluorescence images showing the actin cytoskeleton (Phalloidin-FITC) in podocytes after 6 hours exposure to 8% patient plasma (AB 8/13 passage 28/29/32 (N = 3)). Cells indicated by the red arrow are magnified at the right. (b) Phalloidin-FITC signal intensities were plotted in histogram plots and quantified as shown in (b). *∗∗∗P* < 0.001 compared to the control sample (ctrl). Act. 1 and 2, active 1 and 2; FSGS, focal segmental glomerulosclerosis; Rem, remission.

As mentioned earlier, the detachment and cell death of podocytes after 24 hours exposure to 10% CPF-containing active FSGS plasma was preceded by their shrinking into a round cobblestone-like appearance. Because the actin cytoskeleton in podocytes is largely responsible for their adherent and spread phenotype, we aimed to visualize the actin cytoskeleton after exposure to patient plasma for 6 hours at concentration of 8% because this concentration induces damage but not complete cell death. In response to 8% active FSGS patient plasma with CPF, the podocyte’s actin cytoskeleton was severely disrupted. In fact, most cells had no clear actin cytoskeleton left when compared to podocytes that had been exposed to plasma of the control patient or the FSGS patient that was in remission (Figure 3a). Quantifications of histogram plots displaying the intensity of the actin staining confirmed these observations (Figure 3b).

### CPF-containing Plasma From FSGS Patient Causes Oxidative Stress

**Figure 4:**
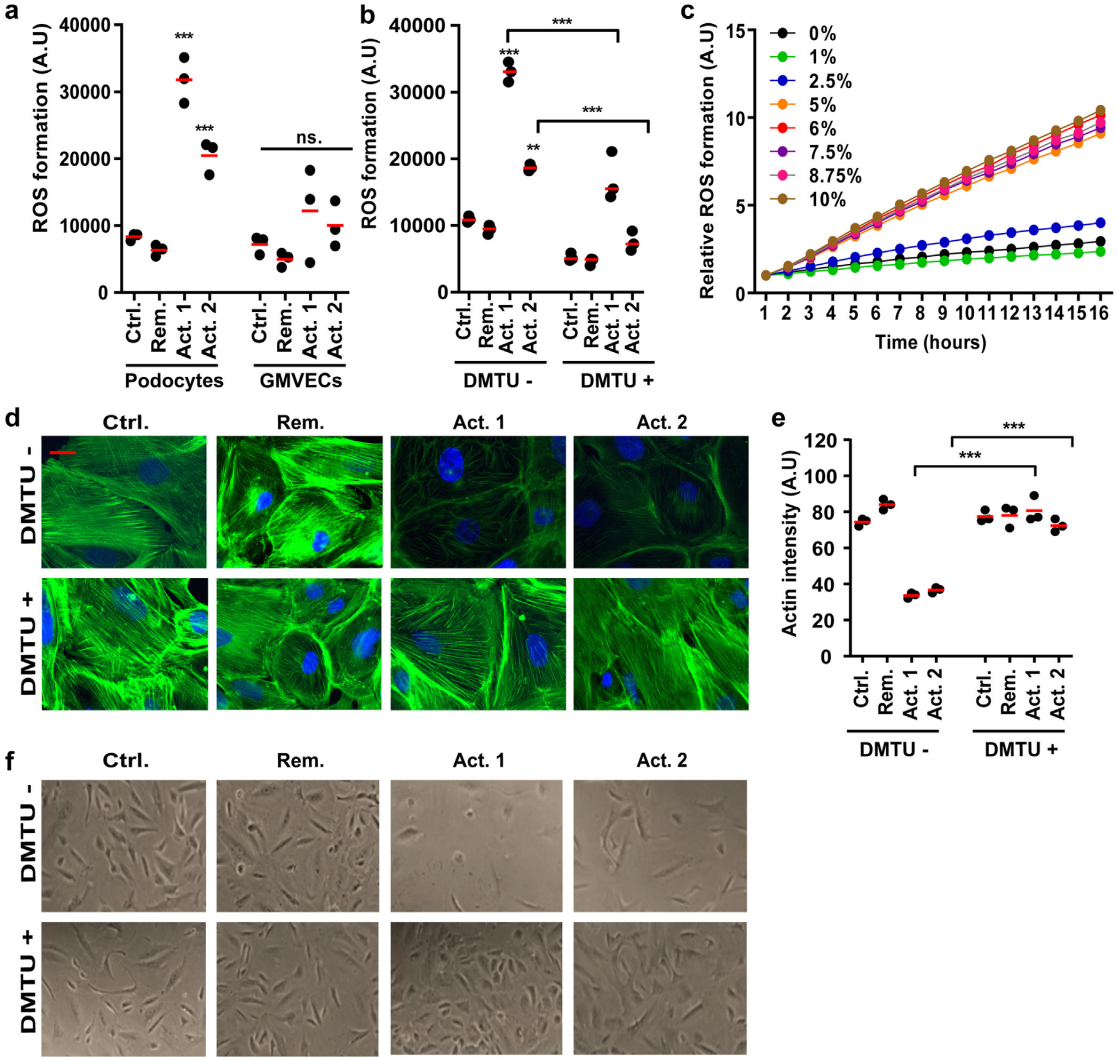
CPF-containing plasma from active FSGS patient causes reactive oxygen species (ROS) formation. (a) ROS formation as detected by CM-H2DCFDA-based fluorometry assays after 24 hours exposure to 8% patient plasma in podocytes and endothelial cells (GMVECs) (N = 3; AB 8/13 passage 20/21/21, GMVECs isolated from 3 different donors with passage 5/5/6). (b) The presence of the ROS scavenger dimethyl thiourea (DMTU; 10 mM) reduces ROS formation in response to 8 % active FSGS patient plasma (Act. 1, Act. 2) (N = 3; AB 8/13 passage 28/29/.33). (c) Kinetics experiments showing ROS formation over time in response to varying concentrations of plasma derived from an active FSGS patient (Act. 2; AB 8/13 passage 20). (d, e) Representative phalloidin-FITC staining of the actin cytoskeleton in podocytes after exposure to 8% patient plasma in the presence or absence of 10 mM DMTU (N = 3; AB 8/13 passage 28/29/33). (e) Green signal intensities from cells were quantified with Adobe Photoshop and are shown in (e). (f) Representative light microscopy images of podocytes after exposure to patient plasma in the presence or absence of 10 mM DMTU. ∗∗*P* < 0.01 and ∗∗∗*P* < 0.001 compared to the control sample (ctrl) and between indicated conditions. Scale bars: 10 μm. Act. 1 and 2, active 1 and 2; DMTU, dimethylthiourea; FSGS, focal segmental glomerulosclerosis; Rem, remission; GMVECs, glomerular microvascular endothelial cells.

Because many cytoskeletal proteins are sensitive to ROS, we hypothesized that CPF plasma-mediated oxidative stress in podocytes is the upstream event of actin degradation.^14^ Indeed, exposure of podocytes to 8% active FSGS patient plasma with a putative CPF caused significant ROS production in podocytes but not in primary GMVECs after 24 hours (Figure 4a). To confirm that we were measuring ROS in our fluorometry assays, we included conditions with the ROS scavenger DMTU. Indeed, the presence of DMTU (10 mM) abolished ROS formation in response to 8% of CPF-containing plasma derived from patients with active FSGS (Figure 4b). Kinetics experiments showed that ROS formation in response to CPF-containing plasma was a fast but gradual process, starting within an hour and gradually increasing over time (Figure 4c). Finally, to evaluate whether ROS formation is actuallly responsible for actin degradation, we stained the actin cytoskeleton after exposure to 8% patient plasma in the presence or absence of 10 mM DMTU. The presence of DMTU could fully prevent actin degradation after exposure to active FSGS patient plasma with CPF for 6 hours (Figure 4d and 4e). By prolonging incubation times to 24 hours, we observed that podocyte cell death was also prevented when DMTU was present (Figure 4f). Taken together, active FSGS patient plasma with CPF causes oxidative stress, which subsequently induces damage to the actin cytoskeleton and results in podocyte death.

### Active FSGS Plasma-Mediated Podocyte Injury Seems to be Caused by the Presence of CPFs

**Figure 5:**
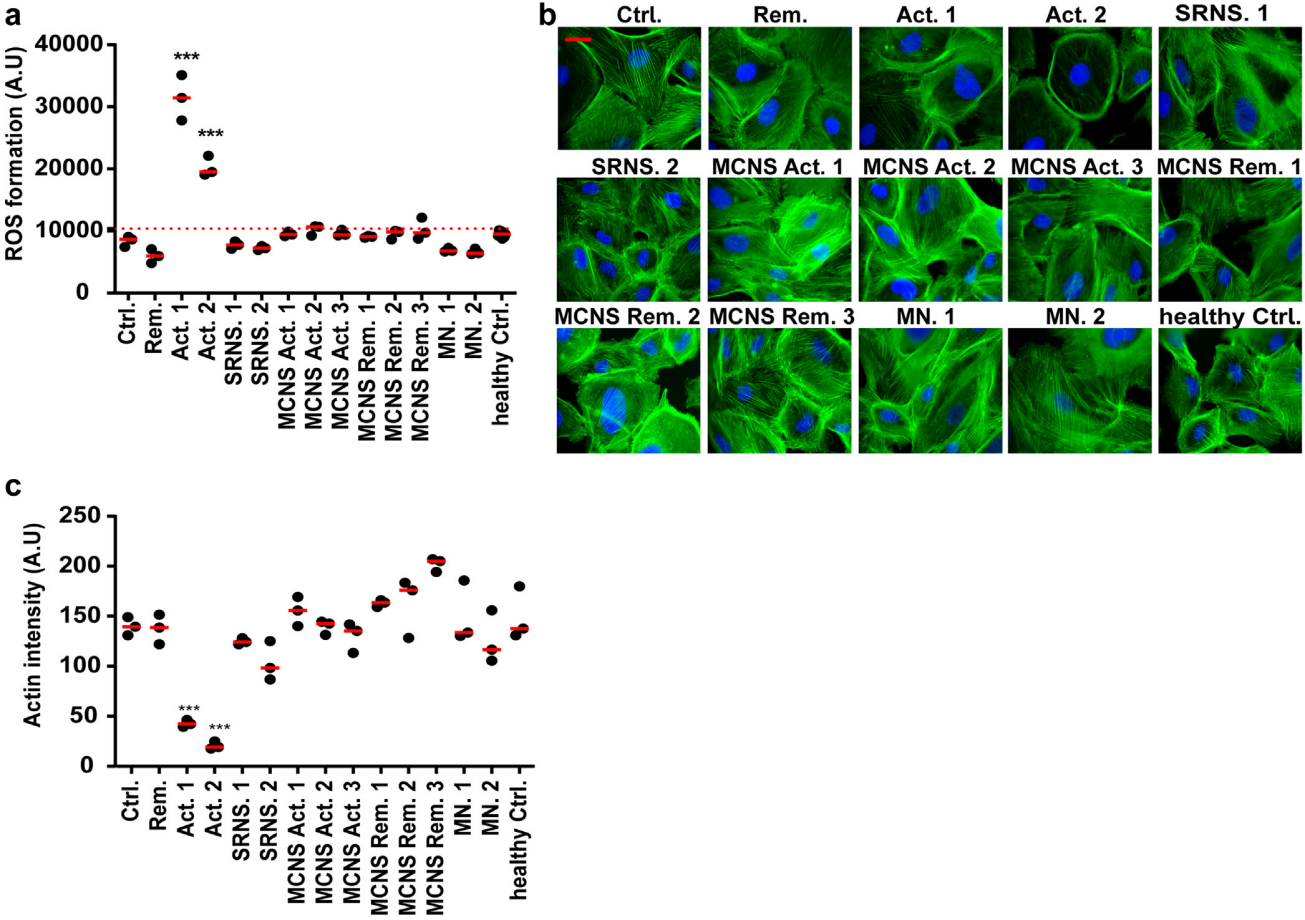
Podocyte injury seems to be mediated by CPF present in the plasma of FSGS patient. (a) Significant ROS formation after 24 hours was observed solely in response to 8% CPF-containing FSGS plasma (b, c) Representative images of phalloidin staining for F-actin in podocytes after exposure to 8% CPF-containing FSGS plasma or 8% sera from patient with other forms of NS (N = 3; AB 8/13 passage 28/29/29). (c) Green signal intensities from cells were quantified with Adobe Photoshop and are shown in (c). *∗∗∗P* < 0.001 compared to the control sample (ctrl). Scale bars: 10 μm. Act. 1 and 2, active 1 and 2; CPF, circulating permeability factor; Ctrl; control (nonkidney patient plasma); FSGS, focal segmental glomerulosclerosis; MCNS, minimal change nephrotic syndrome; MN, membranous nephropathy; Rem, remission; SRNS, steroid-resistant nephrotic syndrome.

We hypothesized that the actin cytoskeleton rearrangement and the ROS production in podocytes are preceded by CPFs present in the plasma of patients with active FSGS. To confirm our hypothesis, we exposed podocytes in addition to the aforementioned samples, to other NS samples and healthy control pooled plasma from the control cohort and measured ROS production and investigated the podocytes’ actin cytoskeleton organization. Significantly increased ROS production was solely seen in response to 8% active FSGS plasma with a putative CPF after 24 hours (Figure 5a). Similarly, significant actin cytoskeleton damage after 6 hours in response to 8% active FSGS plasma with CPF was observed (Figure 5b). Moreover, quantification of the actin signal intensities confirmed that active FSGS plasma containing a putative CPF results in significant podocytes’ actin cytoskeleton injury when compared to other NS samples, a nonkidney control, and healthy control plasma (Figure 5c).

### *In Vitro* Bioassays may be Used to Predict the Presence of a Putative CPF in the Plasma of FSGS Patients

**Figure 6:**
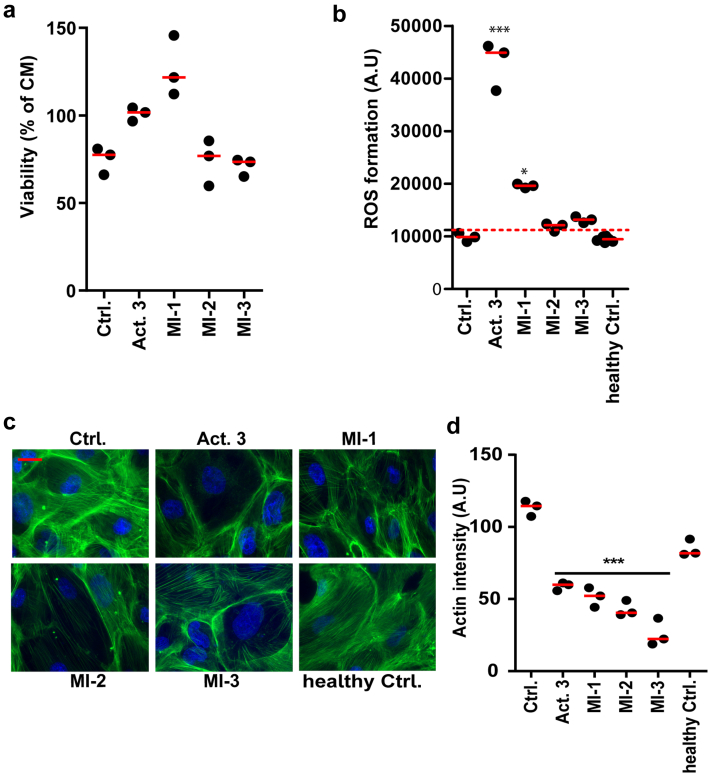
A combination of *in vitro* bioassays may predict the presence of plasma CPF in FSGS patients. (a) Viability assay showing podocytes exposed to 20% active FSGS or rFSGS plasma for 24 hours (N = 3; AB 8/13 passage 27/28/29). (b) ROS formation in podocytes after 24 hours exposure to active FSGS or rFSGS plasma (Act 3 (8 %), MI-1/2/3 (20% plasma) N = 3; AB 8/13 passage 23/24/34). (c) Representative phalloidin-FITC staining of the actin cytoskeleton in podocytes after stimulation with active FSGS or rFSGS plasma (Act. 3 (8 %), MI-1/2/3 (20% plasma) for 6 hours (phalloidin staining with Act. 3 sample was performed N = 3 (AB 8/13 23/27/28) and with samples from Italy N = 1 (due to limited plasma availability) AB 8/13 passage 23). (d) Green actin signal intensities from cells were quantified with Adobe Photoshop and are shown. *∗P* < 0.05 *and ∗∗∗P* < 0.001 compared to the control sample (ctrl). Scale bars: 10 μm. CPF, circulating permeability factor; Ctrl, control (nonkidney patient plasma); FSGS, focal segmental glomerulosclerosis; Rem, remission; Act. 3, active 3; MI-1/2/3, recurrent FSGS (rFSGS) patient plasma from Italy; hCtrl, healthy controls.

Next, we aimed to investigate whether a combination of the ROS assay and the actin cytoskeleton staining assay can identify patients with CPFs in the plasma. For this, 4 additional plasma samples from the validation patient cohort (Table 1) were included. We first measured podocytes’ cell viability after 24 hours exposure to nonkidney control plasma, and the 4 FSGS plasma containing a CPF to determine optimal plasma concentration that induces podocyte injury. Surprisingly, even 20% patient plasma did not cause significant podocyte cell death when compared to a nonkidney control plasma (Figure 6a). ROS production on the other hand was significantly increased after 24 hours when podocytes were exposed to 8% active FSGS plasma (Act. 3) or 20% of MI-1 rFSGS plasma (Figure 6b). In contrast, actin cytoskeleton damage was observed in podocytes in response to all FSGS plasma with a putative CPF (Figure 6c). Quantification of the actin signal intensities indeed confirmed that podocytes exposed to 8% active FSGS plasma or 20% rFSGS plasma resulted in significant actin cytoskeleton degradation (Figure 6d).

## Discussion

Diagnosis of NS patients with a putative CPF is largely based on clinical observations and the interpretation of the treating physician. Unfortunately, the diagnosis of CPF-mediated FSGS is generally made when it is too late (i.e. after a relapse following a kidney transplantation). In this study, using podocyte injury as an experimental readout, we provide a number of assays that demonstrate that the action of CPFs presumed to be present in plasma of patients with FSGS who are responsive to PP can be captured with inexpensive, technically unchallenging, and high-throughput *in vitro* assays. These assays can aid diagnostic or prognostic processes. We show that plasma from patients with FSGS who are responsive to PP, regardless of their kidney function, induces injury to human podocytes *in vitro* as witnessed by the massive generation of ROS, damage to the actin cytoskeleton, and eventually cell death. Such injury is podocyte specific, because human primary glomerular endothelial cells did not show evidence of injury. Furthermore, the ROS production and the actin cytoskeleton damage seemed specific to the presence of a putative CPF in the plasma of FSGS patient because sera from healthy and disease control (SRNS, MCNS, and MN) patients did not cause these effect in podocytes.

Podocyte loss that occurs as a consequence of podocyte injury is a well-known factor in the pathogenesis of FSGS. We observed podocyte cell death in response to 8% or 10% active FSGS plasma but not in response to 5% active FSGS plasma. This seems to indicate that the concentration of a putative CPF in our samples at 5% is too low to cause podocyte cell death. Furthermore, the samples from the validation cohort (rFSGS and Act 3 (active FSGS plasma) merely caused podocyte cell death *in vitro*. Although both podocyte cell death and podocyte detachment result in podocyte loss, the main cause remains yet controversial. Moreover, we do not consider cell viability readout as an appropriate measure to monitor the activity of plasma CPF and therefore only use this readout to determine an optimal concentration to induce podocyte injury in our experiments.

Podocytes are highly dependent on their actin cytoskeleton for many of their functions, because the actin cytoskeleton plays a central role in regulating the podocyte foot processes and filtrating slit diaphragms. Similar to our results, others have shown that plasma from primary FSGS patients and rFSGS patients causes significant disorganization of the podocytes’ actin cytoskeleton.^15^, ^16^, ^17^ In contrary to other studies, however, we show that our actin cytoskeleton staining method can distinguish between CPF-mediated FSGS and other forms of NS, such as SRNS, MCNS, and MN. Moreover, our method is performed in a 96-well cell culture plate and can therefore be easily adapted to an automated high content screening microscopy, which uses hundreds of cells per well to analyze podocyte phenotypes.^14^ Most importantly it does so in an automatic and nonbiased fashion. Some of the drawbacks of our method include visualization of the actin cytoskeleton with fluorescently-labeled phalloidin, which is typically limited to fixed cells because it appears to be cell membrane impermeable and highly toxic.^18^ Furthermore, implementation of high-throughput microscopy techniques, development of image acquisition parameters, and image analysis are still a demanding task.

Podocytes are highly susceptible to ROS, which is shown to cause podocyte foot process effacement. We observed higher levels of ROS production in podocytes exposed to lower concentration (8%) of active FSGS plasma when compared to rFSGS plasma (20%). This is probably due to dilution of “the CPF” concentration by PP rather than treatment modalities such as treatment with immunosuppressive agents. In fact, immunosuppressive therapy has been associated with increased ROS.^19^^,^^20^ Nevertheless, the detection of ROS in podocytes appeared to be a particularly good method for detecting CPF activity in patient plasma. ROS formation in podocytes is likely to be one of the upstream events in the injurious cascade induced by plasma-derived CPFs, and its fluorometric detection with CM-H2DCFDA is a cost effective but highly sensitive method. This assay seems to enable high-throughput screening of CPFs-induced ROS activity and is not influenced by interassay, intra-assay or interobserver variability. Although the measurement of ROS with the CM-H2DCFDA fluorescent probe seemed to be representative of CPF activity, it may not be the most reliable method to measure intracellular ROS production because the range of ROS detected by this probe seems to be broad. Moreover, measurement of ROS with this probe may be influenced by antioxidant enzymes or superoxide that competes with the probe for ROS.^21^ Regardless of these pitfalls, the CM-H2DCFDA probe is a widely used and convenient method to measure general aspects of intracellular ROS and cells’ redox status.

Children with SRNS will generally be screened for genetic mutations, which assists in explaining the cause of the NS, and its management, because immunosuppressive drugs are generally avoided in genetic cases.^22^ Furthermore, finding a genetic cause of the NS will highly reduce the chance of a relapse after kidney transplantation.^23^ Exclusion of a genetic cause is associated with a high chance of FSGS recurrence after transplantation, and until now no tests are available to determine the risk for recurrence. In the absence of such tests, preventive therapies and their potential benefits are difficult to evaluate, whereas preventive therapies for other plasma factors, for instance with ABO incompatible kidney transplantation, have proven to be beneficial.^24^ Our assays may enable early identification of SRNS/FSGS patients with CPFs, who are at a high risk of disease recurrence. Moreover, our assays may also enable better disease management, for example the decision to perform pre-emptive therapeutic interventions such as PP before kidney transplantation.

The role of oxidative stress in the pathogenesis of FSGS has been well established.^25^, ^26^, ^27^, ^28^, ^29^, ^30^ This evidence is derived from both murine and human studies. One of the earliest studies to support the role of oxidative stress involved the injection of hydrogen peroxide in the renal arteries of Munich-Wister rats, which led to rapid and massive proteinuria.^31^ The nicotinamide adenine dinucleotide phosphate oxidase is perceived as the main producer of intracellular ROS.^31^ Indeed, inhibition of nicotinamide adenine dinucleotide phosphate oxidase was shown to ameliorate proteinuria and podocyte foot effacement in mice.^32^ Also, the oral supplementation of the coenzyme Q10 (also known as ubiquinon) to mice was shown to be beneficial in NS by reducing oxidative stress.^33^ In human subjects, evidence supporting a role of oxidative stress in NS is mainly based on the detection of antioxidants and pro-oxidants in blood or urine. In children with NS, circulating pro-oxidants like malondialdehyde and superoxide dismutase have been identified.^34^ Particularly children with active NS, but not those with NS in remission, have high levels of oxidative stress markers.^34^ Other studies have shown that the levels of antioxidants such as plasma protein thiols are decreased in children with active NS but not during remission.^34^

Because the ROS scavenger DMTU was able to ameliorate significant ROS production caused by CPF-containing FSGS plasma, our data may suggest novel therapeutic strategies in FSGS. It seems beneficial to inhibit the intracellular production of ROS or to facilitate rapid scavenging of pro-oxidants. The undisputed antiproteinuric effects of angiotensin-converting enzyme inhibitors and angiotensin II blockers may in part be explained by the inhibition of nicotinamide adenine dinucleotide phosphate oxidase.^35^^,^^36^ Clinically available inhibitors of xanthine oxidase, such as allopurinol or amflutizole, may also have an antiproteinuric effect by reducing ROS production in podocytes.^37^ Scavengers of ROS, such as vitamin E and probucol, have shown beneficial effects in patients with FSGS and MN.^38^, ^39^, ^40^ Calcium antagonists and the beta blocker carvedilol also have ROS scavenging properties;^41^, ^42^, ^43^ yet, whether this effect may play a role in the prevention of oxidative injury in FSGS remains to be determined.

Taking the aforementioned into account, we hypothesize that the *in vitro* detection of ROS in podocytes is a reflection of *in vivo* oxidative stress induced by CPFs circulating in the blood. Interestingly, exposing endothelial cells to patient plasma did not result in the production of ROS formation *in vitro*. This fits with the general conception that FSGS is a disease of the podocyte, even though it is unclear why specifically podocytes encounter oxidative stress in response to plasma of FSGS patients and not endothelial cells. Two possible explanations may be as follows: (i) a responsible receptor that facilitates downstream ROS production is present in podocytes but lacking or less abundantly present on endothelial cells, or (ii) the antioxidant response in endothelial cells is higher compared to that in podocytes. With regard to the first possibility, podocyte-specific receptors that may be involved include angiotensin II receptors or the slit diaphragm-associated protein TRPC6 (transient receptor potential channel C6).^28^^,^^44^ The diminished cytoskeletal actin fibers and the strong cortical actin ring as we observed in podocytes exposed to plasma of patients with active FSGS has previously been shown to be a typical pattern of angiotensin II-mediated cytoskeletal rearrangement in podocytes.^28^^,^^45^ Cytoskeletal rearrangement has been suggested to underlie podocyte foot effacement *in vivo*, which is a crucial early event in the pathophysiology of FSGS.^46^, ^47^, ^48^ Cytoskeletal rearrangement is facilitated by ROS, because the ROS scavenger DMTU prevented disruption of the podocytes’ actin cytoskeleton in our experiments. This ROS-mediated cytoskeleton rearrangement is likely to be dependent on calcium influx and the balance of Rho-GTPases.^49^Interestingly, stimulation of cells with angiotensin II facilitates ROS formation because it activates nicotinamide adenine dinucleotide phosphate oxidase in a protein kinase C-dependent manner.^28^ Of note, podocyte injury in response to angiotensin II is in part mediated by TRPC6.^28^^,^^45^ Gain-of-function mutations in *TRPC6* are well known to be linked with FSGS.^44^ Besides involvement of the renin angiotensin system, the complement system has been also linked to ROS production and consequent podocyte damage. Sublytic complement C5b-9 complex and the complement C3a receptor have been reported to induce ROS production and cause alterations to the actin cytoskeleton of podocytes.^50^^,^^51^

Collectively, this study highlights a deleterious role for oxidative stress in the pathogenesis of FSGS and presents a new framework for tracking *in vivo* CPF activity by measuring plasma-induced ROS formation and assessing the actin cytoskeleton organization in human podocytes *in vitro*. It is undeniable that further studies with larger sample sizes are needed to validate the specificity of the provided assays. Once validated, these assays may provide a specific and noninvasive method to predict the presence of CPFs in FSGS patients. Finally, future studies should focus on the involved key signaling pathways underlying CPF-induced ROS formation and podocyte damage, because such studies may reveal the true identity of the CPF(s) involved.

## Disclosure

All the authors declared no competing interests.
